# Supplementary material for: Brachytherapy Approach Using 177Lu Conjugated Gold Nanostars and Evaluation of Biodistribution, Tumor Retention, Dosimetry and Therapeutic Efficacy in Head and Neck Tumor Model
Source: Pharmaceutics. 2021 Nov 9;13(11):1903. doi: 10.3390/pharmaceutics13111903 (PMC8623985; doi:10.3390/pharmaceutics13111903)
Supplement: Supplementary file 1 [file pharmaceutics-13-01903-s001.zip › pharmaceutics-1433391-supplementary.pdf]

pharmaceutics

Supplementary Materials

Brachytherapy Approach Using ^{177}Lu Conjugated Gold Nanostars and Evaluation of Biodistribution, Tumor Retention, Dosimetry and Therapeutic Efficacy in Head and Neck Tumor Model

Min-Ying Lin [1,†], Hsin-Hua Hsieh [1,†], Jyh-Cheng Chen [1], Chuan-Lin Chen [1], Nin-Chu Sheu [1], Wen-Sheng Huang [2], Shinn-Ying Ho [3], Ting-Wen Chen [3,4], Yi-Jang Lee [1,5,*] and Chun-Yi Wu [1,*]

[1] Department of Biomedical Imaging and Radiological Sciences, National Yang Ming Chiao Tung University, Taipei Branch, Taipei 112, Taiwan; milo841120.be09@nycu.edu.tw (M.-Y.L.); alexa.be09@nycu.edu.tw (H.-H.H.); jcchen@ym.edu.tw (J.-C.C.); clchen2@ym.edu.tw (C.-L.C.); mir870106.y@nycu.edu.tw (N.-C.S.)
[2] Department of Nuclear Medicine, Taipei Medical University Hospital, Taipei 11031, Taiwan; wshuang01@gmail.com
[3] Institute of Bioinformatics and Systems Biology, National Yang Ming Chiao Tung University, Hsinchu Branch, Hsinchu 30068, Taiwan; syho@nctu.edu.tw (S.-Y.H.); dodochen@nctu.edu.tw (T.-W.C.)
[4] Department of Biological Science and Technology, National Yang Ming Chiao Tung University, Hsinchu Branch, Hsinchu 30068, Taiwan
[5] Cancer Progression Research Center, National Yang Ming Chiao Tung University, Taipei Branch, Taipei 112, Taiwan
* Correspondence: yjlee2@nycu.edu.tw (Y.-J.L.); chunyiwu@nycu.edu.tw (C.-Y.W.); Tel.: +886-2-28267189 (Y.-J.L.); +886-2-28267215 (C.-Y.W.)
† These authors contributed equally.

Citation: Lin, M.-Y.; Hsieh, H.-H.; Chen, J.-C.; Chen, C.-L.; Sheu, N.-C.; Huang, W.-S.; Ho, S.-Y.; Chen, T.-W.; Lee, Y.-J.; Wu, C.-Y. Brachytherapy Approach Using ^{177}Lu Conjugated Gold Nanostars and Evaluation of Biodistribution, Tumor Retention, Dosimetry and Therapeutic Efficacy in Head and Neck Tumor Model. *Pharmaceutics* **2021**, *13*, 1903. https://doi.org/10.3390/pharmaceutics13111903

Academic Editors: Cristina Mariana Uritu, Gabriela Dumitrita Stanciu and Luminita Marin

Received: 8 October 2021
Accepted: 5 November
Published: 9 November 2021

Publisher's Note: MDPI stays neutral with regard to jurisdictional claims in published maps and institutional affiliations.

Copyright: © 2021 by the authors. Licensee MDPI, Basel, Switzerland. This article is an open access article distributed under the terms and conditions of the Creative Commons Attribution (CC BY) license (http://creativecommons.org/licenses/by/4.0/).

Table S1. Biodistribution of intravenously injection of ^{177}Lu-DTPA-AuNS in HNSCC orthotopic tumor model[a].

Organs[b]	24 h	48 h
Blood	0.13±0.00	0.04±0.00
Heart	0.25±0.07	0.16±0.04
Lung	0.52±0.27	0.93±0.97
Liver	50.66±13.84	45.58±2.47
Stomach	0.64±0.25	0.33±0.13
Small int.	2.03±0.89	1.15±0.46
Large int.	0.49±0.15	0.30±0.06
Pancreas	0.24±0.10	0.43±0.14
Spleen	14.89±1.60	24.32±3.53
Kidney	6.22±1.21	4.42±0.20
Muscle	0.09±0.04	0.07±0.05
Tumor	1.17±0.13	0.59±0.36
Bone	0.52±0.09	0.66±0.14
Bone Marrow	2.20±0.23	3.38±0.68
Brain	0.01±0.00	0.01±0.01
T/M	14.30±4.86	7.90±0.06
T/B	8.84±1.22	14.52±9.77

a. Unit: percent of injection dose/gram of organ or tissue (%ID/g).

b. T/M: tumor-to-muscle; T/B tumor-to-blood ratio.

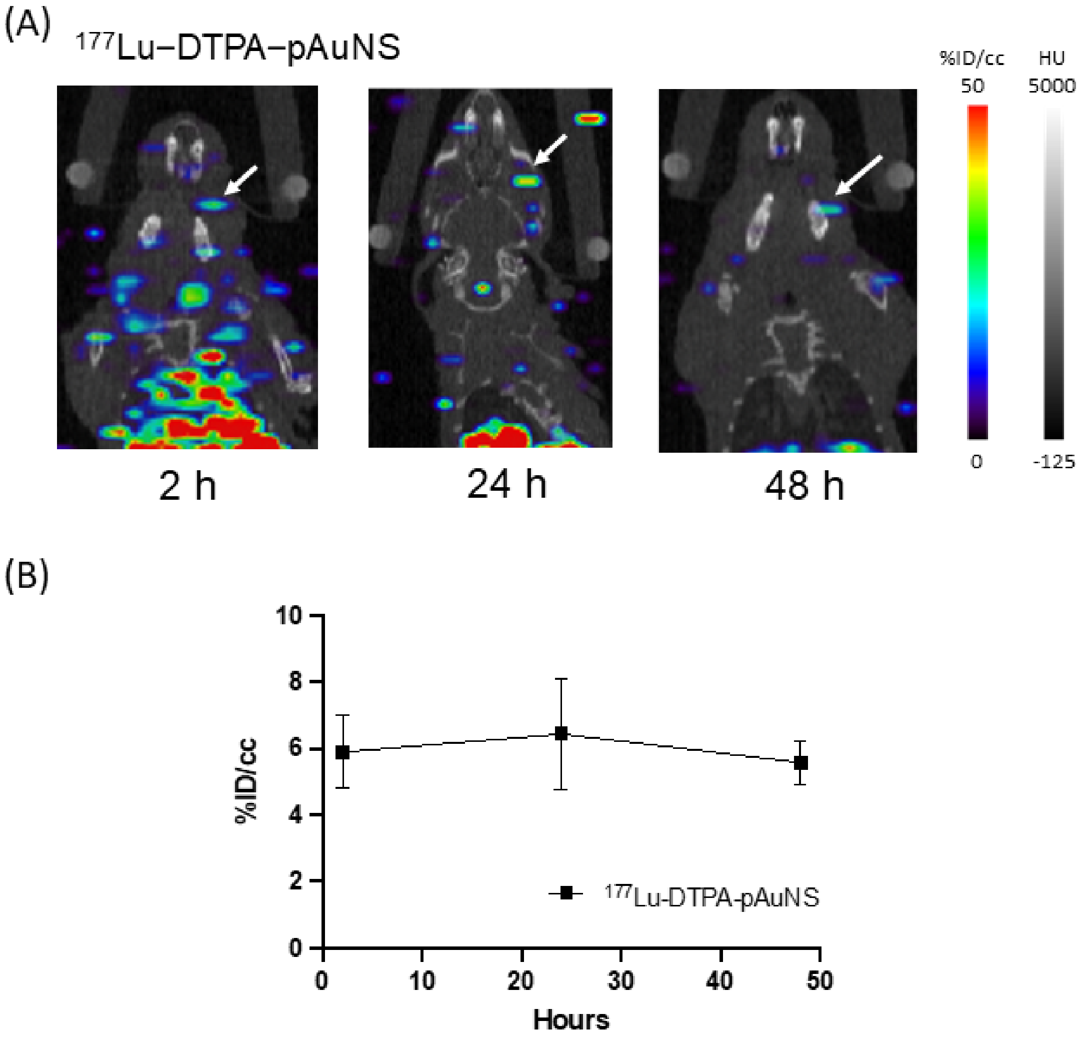

Figure S1. Intravenously injection of ^{177}Lu-DTPA-pAuNS in HNSCC orthotopic tumor model. (**A**) μSPECT/CT imaging of ^{177}Lu-DTPA-pAuNS. (**B**) The activity of the tumor area at different time points.

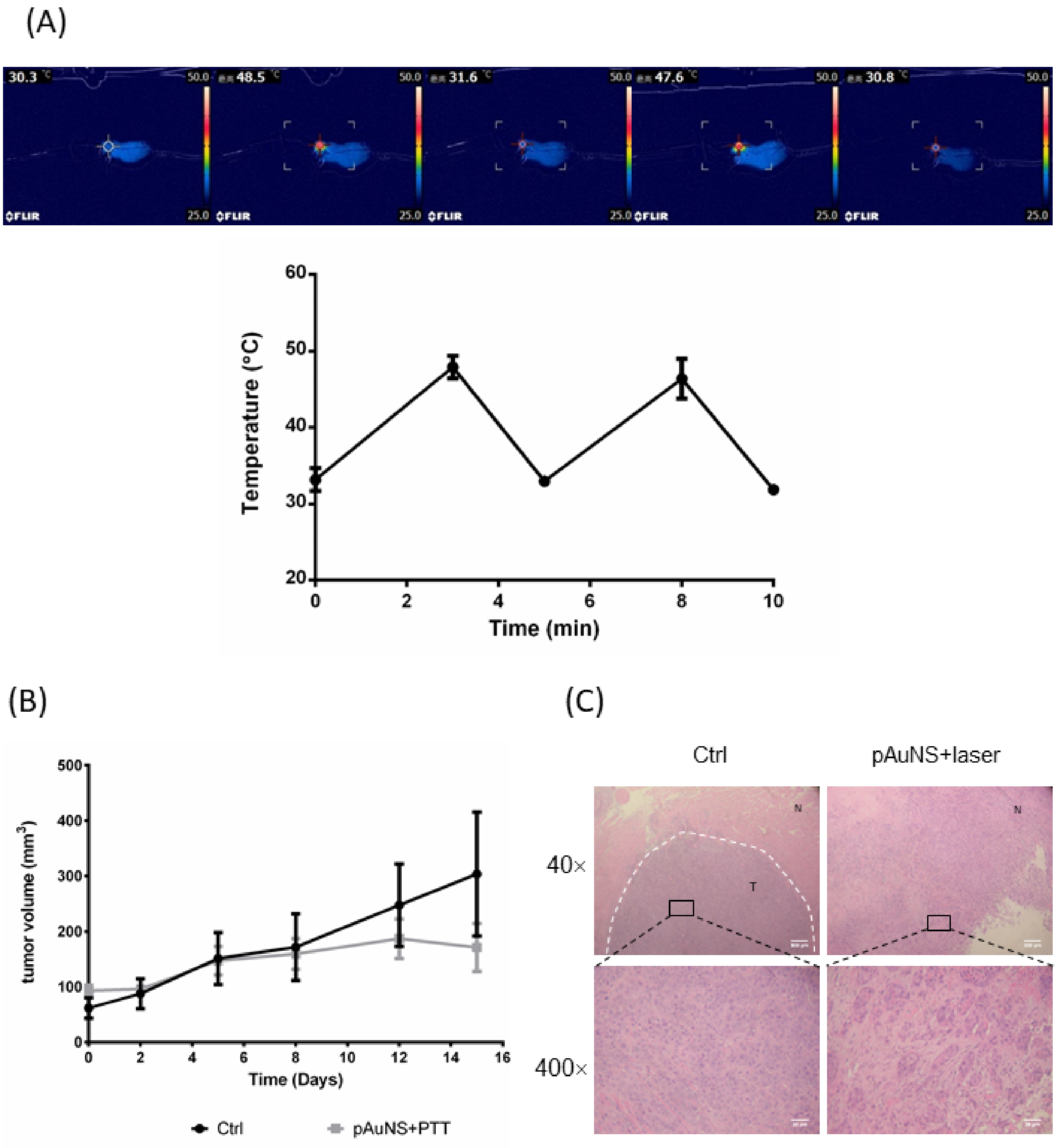

Figure S2. Intratumorally delivered pAuNS-mediated photothermal therapy. (**A**) Thermal imaging of pAuNS-mediated photothermal therapy (PTT) showing the surface temperature of the tumor during laser irradiation. (**B**) The tumor volume curves of the mice received pAuNS-mediated PTT and controls. (**C**) H&E staining of residual masses receiving laser alone (left column) and pAuNS-mediated PTT (right column). The nuclei and the cytoplasm were stained with hematoxylin (blue) and eosin (red). T: tumor; N: non-tumoral surrounding tissues.
